# Characterization of Non-Trivial Neighborhood Fold Constraints from Protein Sequences using Generalized Topohydrophobicity

**DOI:** 10.4137/bbi.s426

**Published:** 2008-01-31

**Authors:** Guillaume Fourty, Isabelle Callebaut, Jean-Paul Mornon

**Affiliations:** Département de Biologie Structurale, Institut de Minéralogie et de Physique des Milieux Condensés (IMPMC), CNRS UMR 7590 — Universités Paris 6/Paris 7, France

**Keywords:** long-range contact, solvent accessibility, multiple alignment, sequence profile, hydrophobicity, regular secondary structures

## Abstract

Prediction of key features of protein structures, such as secondary structure, solvent accessibility and number of contacts between residues, provides useful structural constraints for comparative modeling, fold recognition, *ab-initio* fold prediction and detection of remote relationships. In this study, we aim at characterizing the number of non-trivial close neighbors, or long-range contacts of a residue, as a function of its “topohydrophobic” index deduced from multiple sequence alignments and of the secondary structure in which it is embedded. The “topohydrophobic” index is calculated using a two-class distribution of amino acids, based on their mean atom depths. From a large set of structural alignments processed from the FSSP database, we selected 1485 structural sub-families including at least 8 members, with accurate alignments and limited redundancy. We show that residues within helices, even when deeply buried, have few non-trivial neighbors (0–2), whereas β-strand residues clearly exhibit a multimodal behavior, dominated by the local geometry of the tetrahedron (3 non-trivial close neighbors associated with one tetrahedron; 6 with two tetrahedra). This observed behavior allows the distinction, from sequence profiles, between edge and central β-strands within β-sheets. Useful topological constraints on the immediate neighborhood of an amino acid, but also on its correlated solvent accessibility, can thus be derived using this approach, from the simple knowledge of multiple sequence alignments.

## Introduction

Among the set of relatively simple principles that governs the three-dimensional structures of globular protein domains (Chothia, 1984), two are of obvious importance: i) the masking of a large part of the main chain polarity through the establishment of hydrogen bonds between the amide protons and carbonyl oxygens (mainly within α-helices and β-sheets) and, ii) the hydrophobic effect, underlying the formation of hydrophobic cores of globular domains. In this context, we have highlighted several years ago that strong hydrophobicity has to be conserved in some key positions of a given fold, which were called “topohydrophobic” positions (Poupon and Mornon, 1998; Poupon and Mornon, 1999; Poupon and Mornon, 1999; Poupon and Mornon, 2001). Within a typical globular domain, a third of amino acids belongs to a clear hydrophobic group (VILFMYW), but only a half of these strong hydrophobic amino acids occupies “topohydrophobic” positions (Poupon and Mornon, 1998; Poupon and Mornon, 1999; Poupon and Mornon, 1999; Poupon and Mornon, 2001), which are mainly located within α- and β-regular secondary structures.

“Topohydrophobic” positions have noticeable features, as observed from a comprehensive analysis of structural alignments and their associated three-dimensional structures: i) the amino acids in these positions are much more buried than those occupying “non-topohydrophobic” positions (Poupon and Mornon, 1998); ii) the side chains of these amino acids are markedly less dispersed from one domain to another (though belonging to the same fold), than those located at “non-topohydrophobic” positions (Poupon and Mornon, 1998; Poupon and Mornon, 1999); iii) they constitute a continuous network of positions in close contact, matching well the inner part of the hydrophobic core (Poupon and Mornon, 1998; Poupon and Mornon, 1999); iv) they are mainly occupied by amino acids constituting the folding nuclei (Poupon and Mornon, 1999).

Identification of these “topohydrophobic” positions from the knowledge of sequence data only is possible in practice if an accurate alignment of a small number (e.g. 5 to 8) of sufficiently divergent sequences sharing the same fold (e.g. in the 15–25% sequence identity range) can be performed. From sequence data only, amino acids of crucial importance for the considered fold can be thus highlighted, thereby providing topological constraints at long distance along the sequences, which can be useful in a general way to understand topological features of the protein universe (Lindorff-Larsen et al. 2005).

In the present study, we refine and extend the concept of “topohydrophobic” positions, by introducing a *generalized topohydrophobic index*, which evaluates at each position of a given sequence alignment the fraction of amino acids belonging to the hydrophobic group. We then wish to characterize the number of non-trivial close neighbors of each position of a multiple alignment, depending on this generalized topohydrophobic index deduced from current evolutionary profiles and on the associated predicted secondary structure state. The non-trivial close neighborhood of a residue, which can also be defined as non-local or long range contacts, is the set of amino acids sufficiently distant in the 1D sequence but close in the tertiary structure of the considered protein domain. Residues known to be in local proximity (e.g. covalence and α or β local chain neighbors) are excluded from this set.

In order to define the foundations for predictive studies, we first perform a comprehensive analysis on the basis of accurate reference alignments, selected from structural databases. Hence, we consider a large set of structural alignments allowing good statistics and only focusing on regular secondary structures that are at the building blocks of protein globular domains. Thus, the core blocks defined in this way only include regions aligned with maximal reliability. The topohydrophobic index is based on the natural partition of amino acids in two groups, considering the mean atom depth associated with each kind of amino acid (Pintar et al. 2003; Pintar et al. 2003). This value is indeed closely related to the mean hydrophobicity, and provides a clear separation between hydrophobic residues and the other ones.

The present analysis significantly differs from previous estimations of absolute contact numbers of residues from amino acid sequence data (Fariselli and Casadio, 2000; Ishida et al. 2006; Kinjo et al. 2005; Pollastri et al. 2001; Pollastri et al. 2002; Yuan, 2005). Indeed, these studies generally consider all contacts in a large sphere (typical distance cut-off of 12 Å between Cβ atoms), whereas we focus here on the mean local non-trivial neighborhood of a position within both kinds of regular secondary structures (α-helices and β-strands) using multiple alignments and a short distance cutoff of 7 Å between Cα atoms. Consequently, the number of predicted neighbors is considerably smaller, in the range of 0 to 6, instead of typically 0–50, as described in previous works. Our study also differ from those devoted to the prediction of long range contact maps (e.g. Punta and Rost, 2005), as these do not generally focus on the quantification of these contacts with respect to the secondary structure and to the evolutionary hydrophobicity profile of the considered residue.

We show here that an informative neighborhood of residues can be highlighted from sequence data, which differs between helices (often 0 to 2 such neighbors) and strands (mainly 3 to 6 neighbors). Moreover, a clear multimodal behavior of strands can be observed, with a first main state around three neighbors (tetrahedral arrangement), and the other one around six neighbors (two tetrahedra sharing a vertex). This multimodal behavior allows the distinction between central and edge β-strands. Given the high accuracy reached by secondary structure predictors using multiple alignments (e.g. Frishman and Argos, 1997; Jones, 1999; Pollastri and McLysaght, 2005; Rost and Sander, 1995; Thompson and Goldstein, 1997), the present study offers the possibility of acquiring a good quality information to predict tertiary structures from sequence data only, using a minimal number of parameters.

## Methods

### Datasets and reduction of redundancy

The structural alignments used in this study provide enough data to obtain accurate results, while still supporting a structural relevance. Structural alignments performed and/or extensively corrected by human expertise, as those used for the previous description of “topohydrophobic” positions (Poupon and Mornon, 1998), furnish particular good data; however, due to the considerable increase of structural data, such an expert-based procedure is now unconceivable for analysis on a large scale.

**Figure 1:**
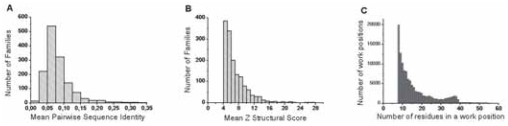
**General features of the FSSP sub-families. A.** Mean pairwise sequence identity calculated on final files within each sub-family. **B.** Distribution of mean structural Z-scores within each FSSP sub-family. **C.** Size distribution of FSSP aligned families. The peak between 30 and 40 members per family corresponds to the existence of fold superfamilies (e.g. the terpenoid synthase superfamily (1di1A)).

Among the main available databases of structural alignments (e.g. BaliBASE (Thompson et al. 1999; Thompson et al. 1999), HOMSTRAD (Mizuguchi et al. 1998), PALI (Balaji et al. 2001), FSSP (Holm and Sander, 1994)), only FSSP (after Families of Structurally Similar Proteins) offers a large number of families, which include at least 8 members and display enough sequence divergence to be informative. For example, PALI, using the SCOP classification (Murzin et al. 1995), only includes, at the time of this study, 171 families with 8 members or more. Moreover, this number dramatically decreases when adding a sequence divergence criterion (Sequence Identity (SI) between two members belonging to a same family shall be less than 50%). FSSP is based on an automatic processing of structural alignments, using a score of structural similarity (Z-score) (Holm and Sander, 1993). The FSSP release we considered contains 2859 sub-families, 2520 being composed of at least 8 members and thus satisfying the selection criteria on work positions, as defined below (Fig. 1). The amount of data is important, as these 2520 alignments include 403 500 sequences, built from 26 577 different amino acid chains. Many chains are therefore present in several sub-families, particularly owing to the presence of the same globular folds within multi-domain proteins. This redundancy has to be reduced before any analysis. To that aim, we use two criteria: the level of sequence identity (SI) and the structural alignment quality (Z). One expects, as a main feature, that the structural quality is on average markedly better within regular secondary structures (α-helices and β-strands) than within coil regions. Hence, we do not consider loops and linker regions, in which alignments are known to be often of bad quality or even senseless.

**Figure 2:**
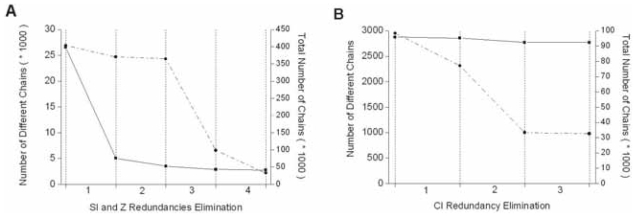
**Redundancy elimination. A.** Evolution of the protein chain numbers: number of different chains (solid line), total number of chains (dotted lines). Step 1; 90 % sequence identity threshold. Step 2; 50 % sequence identity threshold. Step 3; Structural Z-score threshold ≥ 4. Step 4; Composition identity between families ≤ (0.5, 0.5). **B.** The three-steps CI redundancy elimination (see text), number of different chains (solid line), total number of chains (dotted lines).

- **Sequence Identity (SI).** Among families, a pairwise sequence identity (SI) cut-off of 90% dramatically reduces the considered amino acid chain numbers from 26 577 to 5055. A more stringent SI threshold (50%) led to yet conserve 3519 different sequences. We consider this value as a good compromise between the amount of informative data and an acceptable level of redundancy. Meanwhile, the number of families with at least 8 members only slightly decreases (2520 for the initial dataset, 2431 for SI = % and 2406 for SI = 0%). Figure 1A shows that the mean pairwise identity on work positions within each sub-family is indeed low (8.3%), giving evidence for a low redundancy, while keeping good structural superimposition (Fig. 1B).
- **Structural alignment quality (Z). **(Holm and Sander 1994). In the same order of idea, a compromise has to be searched between the amount of data and their structural relevance. Among several thresholds, we choose a low value of Z = 4 for the multiple alignment quality (this value is calculated regarding the leader sequence of the family). Indeed, higher values such as Z ≥ 10 reduce the number of sub-families with at least 8 members to 549, while Z ≥ 4 leads to consider 1721 sub-families. Figure 1B illustrates the actual distribution of Z values (the mean is 7.3), which are in the range of Z-scores between pairs of native-state structural homologues (typically >5 (Dietmann et al. 2002)).
  Combining both thresholds (SI = 50 % and Z ≥ 4), we obtain a database of 1721 sub-families of at least 8 members, including a total of 98 436 sequences, 2876 sequences being distinct from each other. Figure 2A summarizes this process (steps 1 to 3). Step 4 considers a composition identity (CI) threshold between families (0.5, 0.5) (see below and Fig. 2B).
- **Composition identity between families.** On average, each amino acid chain appears in 35 sub-families. Two sub-families may thus contain identical members. This redundancy has also to be reduced as much as possible. To that aim, we compute the composition identity CI_ij_ for each pair (F_i_, F_j_) of N sub-families and consider that they are related if CI_ij_ > D. We then build all the subgroups of related sub-families and, among each subgroup, we eliminate the most common sequences in related families in order to decrease their composition identity to new acceptable CI_ij_ values. This is done until all remaining sub-families in the subgroup are unrelated. Note that if the number of sequences in a given sub-family becomes lower than 8, the sub-family is discarded. Moreover, by eliminating sequences in sub-families that belong to different subgroups, new composition similarities may appear between those sub-families. That is why we decided to perform successive cycles, decreasing the threshold D from 0.8 to the 0.5 final value. During this procedure, we only discard 200 sub-families and 100 amino acid sequences, while two thirds of redundant sequences (approximately 66 000) are eliminated. Figure 2B illustrates the convergence of this process, which leads to a dataset of 1485 sub-families (31 327 sequences and 2727 distinct amino acids chains) with at least 8 members (mean 20) and sharing no more than (0.5,0.5) composition identity (Fig. 1C). In a given family, pairwise sequence identity is necessarily less than 50% and is generally much lower (Fig. 1A) and members have a reliable structural alignment quality (Z ≥ 4) with respect to the leader sequence of the family (mean 7.3, Fig. 1B).

**Figure 3:**
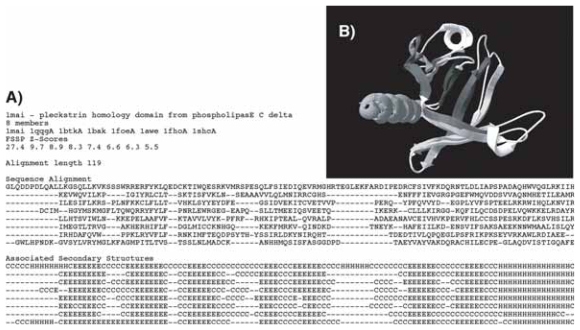
**A sub-family example. A.** Sequence and secondary structure alignment file. The sub-family “1mai”, whose leader sequence is the PH domain of the phospholipase C delta (pdb code 1mai), includes eight members. **B.** Superimposition of the PH folds of 1mai and 1bak (Z-score 8.3), according to the FSSP alignment shown in A. 53 Cα belonging to the seven strands and to the C-terminal helix have been superimposed (RMSD 1.59 Å). The superimposed segments of these two sequences share 19 % of identity (13 % on the entire domain). This superimposition is typical of this sub-family and is representative of the whole bank.

The original FSSP alignments are reformatted according to the following information: sub-family name and PDB accession number of the leader sequence, number of members (≥8), PDB accession numbers of these members, associated structural FSSP Z indexes, alignment length, corresponding aligned sequences and aligned secondary structures (assigned through DSSP (Kabsch and Sander, 1983)). In addition, 3D coordinates of α-Carbons and solvent accessibilities calculated by DSSP (Kabsch and Sander, 1983) are reported for each residue. Figure 3 shows a typical file for a family of eight members.

### Amino acid classes

**Figure 4:**
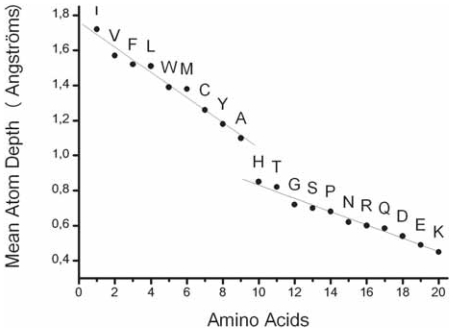
**Mean atom depth.** The original data of Pintar and colleagues (Pintar et al. 2003; Pintar et al. 2003), plotted in the decreasing order of mean atom depths, show two distinct groups of amino acids; on the one hand, the mainly hydrophobic ones (44 % of the total number of amino acids in the bank) and on the other hand neutral and hydrophilic ones (56 % of the amino acids). Histidine, which lies at the frontier between these two groups, was also shown to be the most indifferent amino acid regarding its α beta; or coil states (Callebaut et al. 1997).

The large dataset of reliable multiple alignments constituted here remains however considerably too small to consider the twenty different amino acids in each work position. The clustering of amino acids into a limited number of classes is thus necessary. Usually, three to six classes may be rationally defined (e.g. VILFMYW for the strong hydrophobic class, mainly present within the internal sides of regular secondary structures, GPDSN as main loop-forming residues and ARC-QTEKH for the intermediate class (Callebaut et al. 1997; Hennetin et al. 2003)). Here, we consider a simple partition into two classes, derived from a continuous scaling of the 20 amino acids with respect to their mean atom depth, as defined from a representative set of globular proteins (Pintar et al. 2003; Pintar et al. 2003). Mean atom depth indeed allows the sorting of the 20 amino acids in two distinct groups: IVFLWMCYA (G_1_) and HTGSPNRQDEK (G_2_) (Fig. 4). This classification shows good agreement with mean amino acid burying values, defined through Voronoï tessellations on representative sets of globular domains (Soyer et al. 2000). The two main groups G_1_ (mainly hydrophobic amino acids) and G_2_ (mainly neutral and hydrophilic amino acids) gather 44 and 56% of the total number of amino acids, respectively. The amino acids of group G_1_ are similar to those that were considered hydrophobic by other studies dedicated to long-range contacts (e.g. Punta and Rost, 2005).

### Work positions

We name “work positions” positions in the multiple alignment for which at least 8 amino acids are aligned. The consideration of this absolute number, rather than a relative proportion of all aligned sequences, allows the handling of representative subsets of these alignments, while ignoring positions in which gaps are predominant.

#### Generalized topohydrophobic index

Each work position is characterized by its percentage in amino acids belonging to the G_1_ group. We name it *generalized topohydrophobic index* or *y_1_*, because it records the proportion of hydrophobic amino acids (G_1_) occupying the position. Distributions of the y_1_ parameter are plotted within histograms, according to grouping intervals of 1/8 as a reference to the minimal number of amino acids (8), which have to be present in a work position to be considered.

#### Major secondary structure

**Figure 5:**
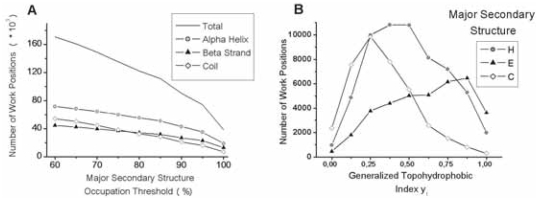
**Work positions. A**. Number of work positions as a function of the percentage of major secondary structure observed at a position of FSSP-derived multiple alignments (x). For x = 75 %, there are 135197 work positions (60021 H, 38860 E, 38316 C). **B**. Populations of the 27 work position types (*see the Results section*) in the final bank with the two groups (G_1_, G_2_) model. H stands for Helix, E for Extended (β—strand) and C for Coil.

We choose to take into account only work positions in which a same secondary structure is sufficiently conserved (at more than x%). Figure 5A shows the number of work positions as a function of this threshold x. We consider that x ≥ 75% offers an acceptable compromise, ensuring that work positions are structurally relevant according to the secondary structure conservation and keeping enough data to perform a large-scale study. Figure 5B shows the distribution of work positions in the different secondary structures as a function of the generalized topohydrophobic index y_1_.

#### Mean solvent accessibility of a work position

Relative accessibilities are computed starting from the absolute accessibilities provided by DSSP (Kabsch and Sander, 1983). The standard accessible surfaces in Å^2^ for residues are derived from canonical G-X-G configuration calculations by Shrake and Rupley (Shrake and Rupley, 1973): A: 124/C: 94/D: 154 E: 187/F: 221/G: 89/H: 201/I: 194/K: 214/L: 198/M: 215/N: 161/P: 150/Q: 190/R: 244/S: 126/T: 152/V: 169/W: 265 and Y: 236. Relative accessibility of a work position is the mean value of the relative accessibilities of its residues.

#### Non-trivial neighbors

**Figure 6:**
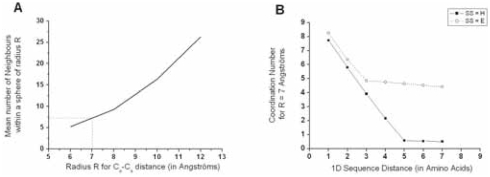
**Neighboring definition. A**. Mean number of neighbors within a sphere of radius R, as a function of Cα-Cα distance R, calculated on the FSSP-derived bank. For R = 7 Å (a value generally retained to characterize close neighborhood of an amino acid), the mean coordination number is between 7 and 8. **B**. Evolution of the mean coordination number for R = 7 Å as a function of the sequence distance D, expressed in amino acids. For D = 1, all contacts are taken into account and the mean values are close from each other for strands (E) or helices (H). Above D = 2, behaviors of strands and helices differ, as strands assemble to form sheets with a high and constant mean number of neighbors (~4.5), while helices only show a small mean value of ~0.5 when D ≥ 4. For the E and H states, we consider that beyond D = 6, neighbors are only non-trivial ones.

The non-trivial neighborhood of an amino acid can be described from the known atomic coordinates. Two amino acids are defined as non-trivial neighbors if their Cα are separated by less than 7 Å (Tudos et al. 1994) and if they are distant in sequence from more than 6 residues (Fig. 6). The mean number of neighbors for a work position is defined as the average number of non-trivial neighbors of the amino acids belonging to that position. An even better way to consider the amino acid neighborhood, which is independent of a cutoff threshold value, would have been to use a description through pondered Voronoï tessellations (Angelov et al. 2002; Dupuis et al. 2005; Dupuis et al. 2004; Soyer et al. 2000). However, this description is prohibitively time-consuming and thus out of scope for a large-scale study.

## Results

### Dataset

A set of benchmark alignments is selected as described in the *Methods* section, in order to estimate the number of long-range (or non trivial) contacts of amino acids, with respect to the *general topohydrophobic index* deduced from the multiple sequence alignment and to the associated secondary structure. The dataset considered here includes 1485 sub-families (31 327 sequences and 2727 distinct amino acids chains) with at least 8 members (mean number 20) and sharing no more than (0.5, 0.5) composition identity, a parameter that was introduced in order to avoid redundancy between subfamilies. In a given family, pairwise sequence identity is necessarily less than 50% and quite always far below (mean 8.3 %) and the members have a confident structural alignment quality (Z) of at least 4 (mean 7.3) with respect of the leader sequence of the family. It is worth noting that all proteins sharing a same fold, fulfilling the selected sequence identity and structural alignment quality criteria described above, are not clustered into a unique family. Some sub-families described above are subsets of proteins possessing at least one domain with a given fold. This distribution in several sub-groups is directly dependent on the initial FSSP dataset and to the selection procedure. For example, some members of the family shown in Figure 3 (family 1mai—Pleckstrin Homology (PH) fold) are found in eight other families with a PH fold domain. However, the alignments well cover the known universe of globular domains, and are thus representative of the structural conservation and diversity within proteins.

We analyze the main features of “work positions” in multiple alignments (see definition in the *Methods* section), for which more than 75% of the residues share the same secondary structure. As structural superimpositions and secondary structure assignments were automatically performed, local mismatches may occur. However, these mismatches only constitute a marginal fraction within the final alignments obtained after filtering of the initial dataset. Only 8% of the 97 000 retained work positions exhibit more than one H/E discrepancy and thus only constitute a background noise, which do not sensibly modify the main results of this study. The good quality of solvent accessibility predictions, which are directly performed on our filtered database of structural alignments (see below) and are similar to results obtained with other methods (Gianese et al. 2003; Pascarella et al. 1998; Rost and Sander, 1994; Thompson and Goldstein, 1996), further supports the overall structural relevance of work positions.

The partition of amino acids in two groups G_1_ (IVFLWMCA) and G_2_ (HTGSPNRQDEK), as introduced in the *Methods* section, and the distribution of group compositions in 1/8 lead to 9 distinct topohydrophobic y_1_ values (0, 0.125, …, 1), which can describe a work position. 27 classes of work positions (X, y_1_) can thus exist, combining y_1_ and X, the major secondary structure (X = helix, strand or coil). The 27 classes are often largely represented in the bank. The less populated classes are the limit cases, consisting in fully hydrophilic strands (Strand, 0) and fully hydrophobic coils (Coil, 1) (462 and 296 work positions, respectively; Fig. 5B). We principally consider the 18 classes of work positions associated with regular secondary structures (X = H or E; 60 021 and 36 830 work positions, respectively).

### Positions within helices

#### Relative solvent accessibility

**Figure 7:**
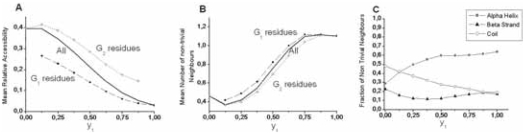
**Helices. A**. Mean solvent accessibility for helices, as a function of the composition of work positions. When positions have a high topohydrophobic index y_1_, the G2 class adopts a similar behavior as the G1 one, constrained by fold requirements. **B**. Evolution of the mean number of non-trivial neighbors as a function of the composition of work positions in α regular secondary structures. The same comment as for A can be made for G2 amino acids. **C.** Partners of helix work positions. When topohydrophobicity is high, Helix-Helix and particularly G_1_-G_1_ contacts dominate in α regular secondary structures.

**Figure 8:**
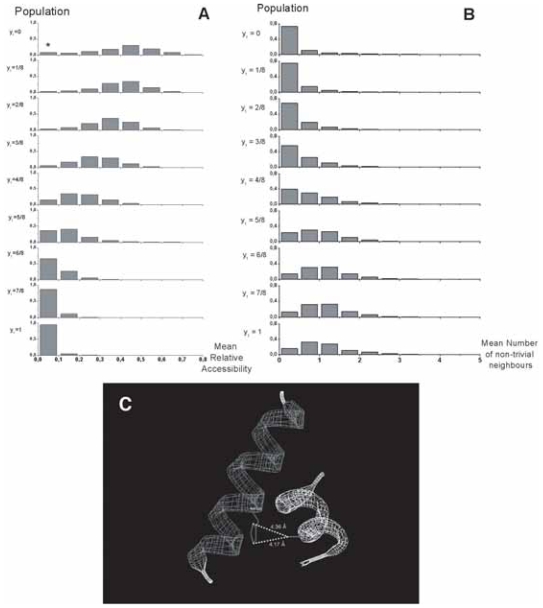
**Helices. A**. Distributions of work positions according to mean relative solvent accessibility and hydrophobicity for α regular secondary structures. Star indicates an exceeding value, likely resulting from salt bridges and mutually neutralizing pairs of hydrophilic amino acids within protein cores. **B**. Distributions of work positions according to the mean number of non-trivial neighbors and hydrophobicity in α regular secondary structures. **C**. Typical single inter-helix contact found in 1mai, between H120 and A21.

Figure 7A illustrates the behavior of the mean relative solvent accessibility in helix work positions within multiple alignments, as a function of the generalized topohydrophobic index y_1_, ranging from 0 to 1. As expected, the mean relative accessibility to solvent diminishes when y_1_ increases. We also consider the individual behaviors of G_1_- and G_2_-residues. We observe that the G_1_- and G_2_-values depend on the y_1_ value of the work positions, and both diminish when y_1_ increases. The two curves are quite parallel for the two groups, with the G_1_ mean values smaller, as expected, than the G_2_ ones. The distribution of mean relative accessibilities around the mean values, shown in Figure 7A, is illustrated in Figure 8A. For very low y_1_ values (low hydrophobicity), the mean relative accessibilities are distributed according to a Gaussian-like rule centered on 0.45 and, as y_1_ increases, this curve smashes towards the origin, with a mean below 0.1 for 95% of the 1977 totally hydrophobic work positions (y_1_ = 1). For y_1_ = 0 (fully neutral or hydrophilic positions), a small peak, indicated by a star, reveals the existence of buried positions. It likely corresponds to salt bridges, and more generally to pairs of side-chains in mutual neutralizing polar contacts within globular cores. This observation moreover provides indirect biophysical support to the data quality of the FSSP-derived bank.

#### Number of non-trivial close neighbors

The number of non-trivial close neighbors (Fig. 8B) shows a symmetrical behavior compared to the relative accessibility (Fig. 8A). The number of non-trivial neighbors of work positions within helices increases as hydrophobicity rises from y_1_ = 0 to y_1_ = 1, but is rarely greater than 2, even for completely buried positions (mean accessibility < 0.1), within the internal sides of helices. This mainly results from the principal occupancy, in such configurations, of the close neighborhood by trivial neighbors, which restrains the free space for external residues, and from the convex geometry of α-helices, roughly cylindrical, with a large dispersion of side chains. G_1_ and G_2_ groups are both concerned by this increase of the number of nontrivial neighbors (Fig. 7B). Work positions with high hydrophobicity within helices mainly establish contacts with other helices (Fig. 7C). Moreover, these contacts mainly involve G_1_ amino acids within the hydrophobic core (data not shown). Figure 8C illustrates such a situation.

### Positions within strands

**Figure 9:**
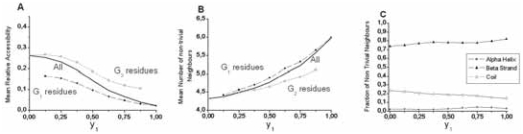
**Strands. A**. Evolution of the mean relative solvent accessibility for β-strands, as a function of hydrophobicity of work positions. **B**. Evolution of the mean number of non-trivial neighbors; as a function of the composition of β work positions. **C**. Partners of β-strand work positions. At high topohydrophobicity, strand-strand and particularly G1-G1 contacts dominate in β-sheets.

**Figure 10:**
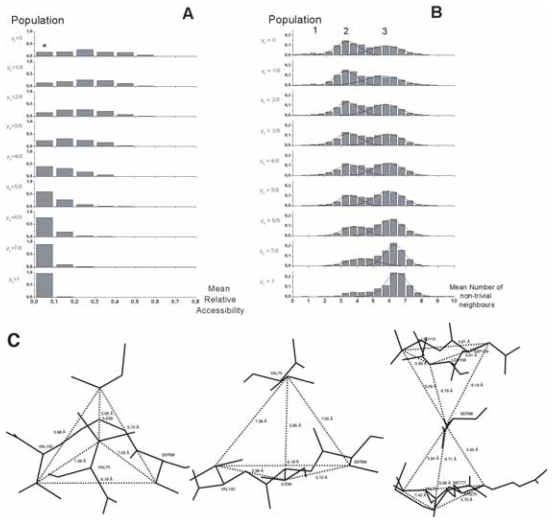
**Strands. A**. Distributions of work positions according to mean relative solvent accessibility and hydrophobicity for β regular secondary structures. The presence of salt bridges and hydrophilic pairs likely account for the value indicated by a star, as for helix positions. **B**. Distributions of work positions according to mean number of non-trivial neighbors and hydrophobicity in β regular secondary structures. Using Gaussian approximation to deconvoluate the overall profile highlights the multimodal distribution of strand neighbors. Three modes (1, 2 and 3) are present: ~1.2, 3.3 to 4.5 and 5.6 to 6.5 mean neighbors, respectively. **C**. Two first views. Current tetrahedron found between Cβ of residues i, i +1, i +2 of a strand and another residue in an adjacent strand. The example shown in two orthogonal views is from 1mai (S98, I99, V100 and V75). The mean tetrahedron edge size is 6.3 Å. Last view. Two tetrahedra sharing a vertex: i, i +1, i +2 of a strand; j, j +1, j +2 of another one, which sandwiches a residue. The shown example is also taken from 1mai (V75, R76, M77/L108, D109, L110/S98; mean edge size of 5.9 Å).

A similar investigation was performed for work positions associated with β-strands (Figs. 9 and 10). The most striking result for β-strands is a strong increase of the number of the non-trivial first neighbors and a clearly multimodal distribution observed for almost all y_1_ values, and in particular for the less hydrophobic ones (low y_1_ values). The weakly populated mode, centered on approximately one neighbor, is likely associated with highly external positions at the extremity of some strands. The two other modes (near 3 and 6 neighbors) are likely to correspond to external (edge) and internal (central) positions of strands within β-sheets, respectively. Indeed, the second mode (around 3) mainly relies on the architecture of β-strands within sheets, where side chains in positions i, i + 1, i + 2 in one strand occupy a roughly equilateral triangle. This triangle constitutes the basis of the interaction with another amino acid j of a neighboring strand, linked to the “i” strand through canonical main chain H-bonds. These four residues constitute a more or less deformed tetrahedron (distance between Cβ ~6.2 Å), which represents the basic unit of compact packing of similar sized spheres (Fig. 10C). The third mode (around 6) mainly corresponds to a geometry with two tetrahedra (one strand sandwiched by two others) sharing a vertex, which has 6 first non-trivial neighbors (Fig. 10C). Many deviations from this ideal scheme occur and tend to flatten the Gaussian distribution. As for helices, the number of non-trivial neighbors increases with hydrophobicity of a work position (Fig. 9B) and strand non-trivial neighbors are very often found within other strands (Fig. 9C). The present study quantifies this behavior and offers the opportunity to gain information on the probable participation of an amino acid in an internal or external strand position, through the only knowledge of multiple sequence alignments.

### Influence of fold classes

**Figure 11:**
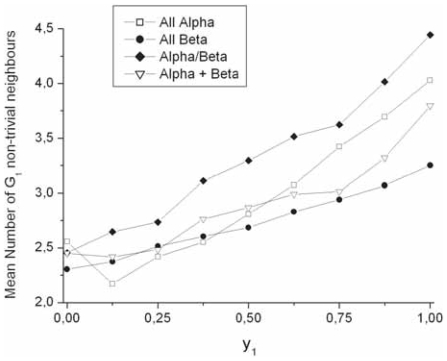
Mean number of observed G_1_ non-trivial neighbors within the main fold classes.

The dataset is large enough to estimate the putative influence of fold classes on some parameters. Four main classes, as described in the SCOP classification (Murzin et al. 1995), were considered (all-α (297 sub-families), all-β (370 sub-families), α/β (530 sub-families) and α + β (131 sub-families)). One can expect that differences in the tertiary structures between the four fold classes are reflected in the level of hydrophobic contacts, involving residues of the G1 group, and in particular in positions with a high topohydrophobic index (y_1_ = 1). Hence, one can observe that the mean number of non-trivial neighbors belonging to the G_1_ group for strand work positions with a high topohydrophobic index is sensibly higher for the α/β class than for the three others (4.51 versus 4.02 (α), 3.25 (β) and 3.79 (α + β); Fig. 11). This is all the more noticeable than the total number of non-trivial neighbors of strands work position with a topohydrophobic index of 1 is rather constant (Table 1). A hypothesis to explain such a behavior is that a larger number of fully hydrophobic work positions with a structural role exist in the α/β and even α classes, but this remains to be further investigated. Furthermore, one can note that better performance of programs for the prediction of long-range contacts are reported by at least two studies for this same α/β class (MacCallum, 2004; Punta and Rost, 2005).

## Discussion

The prediction of non-trivial neighborhood, or long-range contacts, from protein sequences is of particular interest to improve comparative modeling and to enhance fold recognition and *ab-initio* fold prediction. It can also help to detect remote relationships between protein sequences and to solve experimental structures. Contact prediction methods have received much attention during the last decade and often combine the evolutionary information available from multiple alignments and the prediction of secondary structures. They can be roughly classified in two non-exclusive categories: statistical correlated mutations approaches (*see for examples* Halperin et al. 2006; Kundrotas and Alexov, 2006) and machine-learning approaches (*see for example* Punta and Rost, 2005). While most methods aimed at predicting contact maps, several other approaches have been developed to estimate the total number of contacts (Fariselli and Casadio, 2000; Ishida et al. 2006; Kinjo et al. 2005; Pollastri et al. 2001; Pollastri et al. 2002; Yuan, 2005), but these generally define large numbers of coordination, including trivial neighborhood, and rarely link these numbers to the topological and evolutionary features of the region which includes the concerned residue.

Our analysis outlines the relationship between the mean number of non-trivial neighbors and a *topohydrophobic* index, which relies on the mean hydrophobicity of a position within a multiple alignment of sequences, as a function of the secondary structure. The topological data we collected here might be used in a predictive perspective, as secondary structures can currently be predicted with a good accuracy using multiple alignments (see for example Rost and Sander, 1993). As noticed in earlier studies (Punta and Rost, 2005), the performance of the various estimations that can be made on the long-range contacts directly depends on the quality of the evolutionary profiles, which have to be large and to contain divergent sequences to furnish accurate information.

The original result of this study is that different behaviors relative to non-trivial neighbors can be observed for helix and for strand residues, and among strands, for central and edge β-strands. Starting from these observations, the prediction of the topological nature of β-strands can be approached using classification methods like decision trees (see Supplementary data 1). Briefly, using parameters such as the length of the strand, its mean hydrophobicity and periodicity of G_1_ and G_2_ residues, combined with topohydrophobic index, decision trees lead to an accuracy of 80% for the prediction of edge/central positions within β-sheets (Supplementary data 1). Although it is difficult to compare methods using different datasets for training and prediction, this approach appears to achieve a prediction accuracy similar to the one obtained by Siepen and coworkers (Siepen et al. 2003), which is based on the use of support vector machine (SVM) and decision trees.

The use of the topohydrophobic index, combined with information on the nature of secondary structures, the group (G_1_ or G_2_) to which the residue belongs, as well as environmental parameters, describing the local periodicity, also allows the prediction of the relative solvent accessibilities of a residue within a work position into two or three states models (exposed, intermediate and buried; see Supplementary data 2). In the ideal case, when secondary structures are “known”, solvent accessibility predictions using this methodology led to Q2 of 79% (16% threshold) versus 75% for other methods tested on the same dataset and based on neural networks (Rost and Sander 1994) or probability profiles/support vector machines (Gianese et al. 2003) and to Q3 of 65% versus 58% for the same other methods (9–36 % threshold). On the one hand, the accurate prediction of solvent accessibility using generalized “topohydrophobicity” provides additional constraints on informative positions of a sequence (the work positions). On the other hand, these results further support the intrinsic quality of the dataset used for this study.

The present analysis shed light on important geometrical and topological parameters that can help to understand protein sequence-fold relationships. It appears of particular interest that the dichotomy (hydrophobicity—hydrophilicity) between only two nearly equally populated classes of amino acids provides a very simple way to derive useful and often accurate topological data, that can be useful for protein fold recognition.

## Supplementary Material

### Supplementary Data 1

#### Use of decision trees for predicting the edge/central nature of β-strands, as a function of the topohydrophobic index and of the predicted secondary structures

Using sequence data from work positions in multiple alignments and the J4.8 implementation of the C4.5 program (Quinlan, 1993) to derive decision trees, we aimed at predicting the topological nature of strands (central, or edge). We adopted the following strategy:

##### Dataset

We used information provided by DSSP (Kabsch and Sander, 1983) on beta partners (BP) and we only considered “complete” strands (undamaged by the DSSP assignment and the FSSP automatic multiple alignment procedure). We identified on the leader sequences of the 1485 sub-families, 7541 central strands and 7886 edge strands (49% and 51% of the total strands, respectively). We observed that 75% of amino acids possessing less than four non-trivial neighbors belong to edge strands, while 83% of amino acids possessing more than five non-trivial neighbors are central strands. This can be related to the canonical neighborhood of one or two tetrahedral configurations, as commented in the main text. Among the 15407 strands selected above, 8018 possess at least one well-defined work position, which can thus be used to predict their nature (central or edge). This dataset was used to provide training and cross-validation data.

##### Selected attributes for decision tree classification

We used only few attributes in order to obtain a simple classification tree (and therefore simple rules) and to easily discriminate their influence in the prediction process. The parameters were the *length L of the strand* expressed in amino acid units, the *strand hydrophobicity H* defined as H = ∑^L^ _i=1_ h_i_/L (h_i_ = 1 for G1 amino acids and 0 for G2 ones), the *polar periodicity of the strand P* defined as P = ∑ p_i_/(L−1) (p_i_= 0 if h_i_ = h_i+1_ and p_i_=1 if h_i_ ≠ h_i+1_), the *strand charge C* defined as C = ∑ C_i_/L (C_i_ = 1 for D, E, K, R, H, C_i_ = 0 for other amino acids). These parameters can be extended to the mean values H_m_, P_m_, C_m_ for aligned sequences within sub-families. From multiple sequence alignments, we also introduced a simple additional parameter: the mean topohydrophobicity, T_m_, which is the mean of y_1_ indexes when several work positions are present in the considered strand.

**Figure S1:**
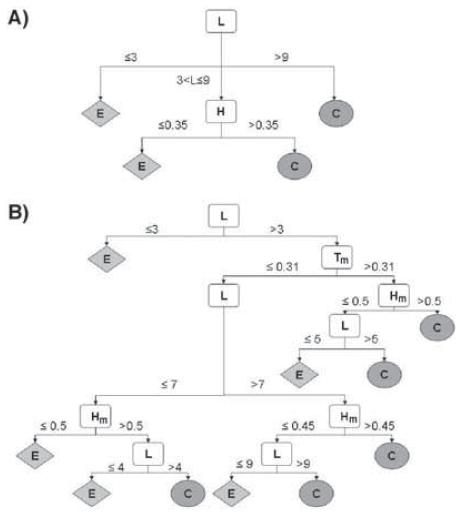
Decision trees for predicting the edge (E) or central (C) position of β-strands A) The simplest decision tree, leading to 77% of good predictions. B) A more complex tree giving 80% of good predictions. L: length of the β-strand; H: hydrophobicity of the β-strand; T: topohydrophobicity. The m index stands for mean values.

The predictive power of this approach shall be compared to the basic level of a random prediction (50%) or that of the major class (edge β-strands) at 51%. Table S1 shows the results for the leader strands of the considered sub-families, using various decision trees built with single parameters or combinations of them. Immediately after the length L, hydrophobicity H is determinant. With only two parameters, a decision tree is yet efficient to distinguish central and edge strands, as shown in Figure S1 and gives 77% of good predictions. The use of the strand length L and two parameters deduced from multiple sequence alignments H_m_ and T_m_ leads to nearly 80% of good predictions (Table S2). This combination seems to be the best one, although implying only few basic data. As often, it is difficult to precisely compare these results with other approaches dealing with the same topic, as many features differ. However, prediction accuracy appears to reach the same level as in a previous study (Siepen et al. 2003). This analysis uses secondary structure elements (β-strands) defined using DSSP (Kabsch and Sander, 1983) from experimental structures. Accuracy should be reduced starting from secondary structure predictions, although a good level of secondary structure prediction accuracy can now be reached using predictive tools such as PSI-PRED (Jones, 1999).

**Table S1:** Prediction results from several combinations of attributes.

Attributes	Good predictions	r
L	72%	0,44
H	68%	0,36
P	66%	0,33
C	64%	0,28
L+H	77%	0,53
L+C	74%	0,48
L+P	73%	0,46
C+H	72%	0,44
L+H+C	77%	0,53
L+H+C+P	77%	0,54

r corresponds to the Matthews correlation coefficient (MCC) for two states (Matthews, 1975)

**Table S2:** Predictions associated to selected combinations of attributes (see text).

Attributes	Q_2_	r
L+H_m_	79%	0,58
L+H_m_+C_m_	79%	0,59
L+H_m_+T_m_	80%	0,60

r corresponds to the Matthews correlation coefficient (MCC) for two states (Matthews, 1975)

ReferencesJonesDT1999Protein secondary structure prediction based on position-specific scoring matricesJ. Mol. Biol2921952021049386810.1006/jmbi.1999.3091KabschWSanderC1983Dictionary of protein secondary structure: pattern recognition of hydrogen-bonded and geometrical featuresBiopolymers222577637666733310.1002/bip.360221211MatthewsBW1975Comparison of the predicted and observed secondary structure of T4 phage lysozymeBiochim Biophys. Acta40544251118096710.1016/0005-2795(75)90109-9SiepenJARadfordSEWestheadDR2003Beta edge strands in protein structure prediction and aggregationProtein Sci122348591450089310.1110/ps.03234503PMC2366916

### Supplementary Data 2

#### Use of decision trees for predicting the relative accessibility as a function of the topohydrophobic index and of the predicted secondary structures

The estimation of the number of non-trivial neighbors described in this study is based on divergent and accurate multiple sequence alignments, explored through a highly simplified alphabet made of only two amino acid classes G_1_ and G_2_ (*see main text*). We similarly addressed the prediction of the relative solvent accessibility of a residue into two or three state models. First, in order to calibrate the process, we considered that the secondary structures are known, i.e. we used the DSSP assignments based on 3D coordinates (Kabsch and Sander, 1983). Then, we used this approach to predict the burying of selected positions within the multiple alignments, assuming that secondary structure predictions in these positions are accurate.

##### Selected attributes for the decision tree classification

To describe a residue of the leader sequence, occupying a work position, we used the *secondary structure state* (H or E), the *generalized topohydrophobic parameter y*_1_ deduced from the multiple sequence alignment, the *group parameter G_1_* (0 or 1) of the considered residue and *four environment parameters EnvIn reply to: (i= 1, 4)* associated with positions i. EnvIn reply to: = (G_n−i_ + G_n+i_)/2. Env_i_ can thus take three values: 1.0, 0.5, 0.0, describing the local periodicity. Gaps and 5 amino acids at each extremity of the sequence were discarded. As for the prediction of edge and central β-strands, these attributes were completed by those derived from multiple alignments, which are the SSM (Secondary Structure — Major state) and the topohydrophobic index y_1_. Building decision trees with those 7 attributes is time consuming when studying the whole FSSP-derived database. In order to overcome this difficulty, we used a reduced bank of 270 multiple alignments derived from the 1485 sub-families of the whole bank. These 270 leader sequences include non-redundant SCOP folds with a total of 77 108 amino acids and 16 000 (H or E) work positions.

**Figure S2:**
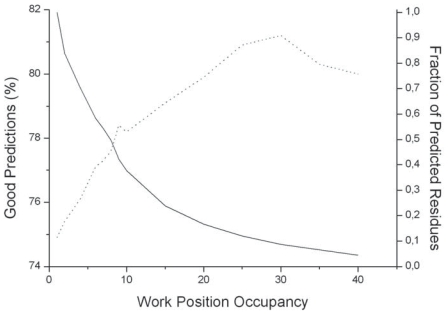
**Prediction of relative solvent accessibility.** Evolution of the level of good predictions (dotted line) and of the fraction of predicted residues (solid line), as a function of the occupancy of work positions. The accessibility threshold is fixed to 16% and the secondary structure conservation to 75%.

**Figure S3:**
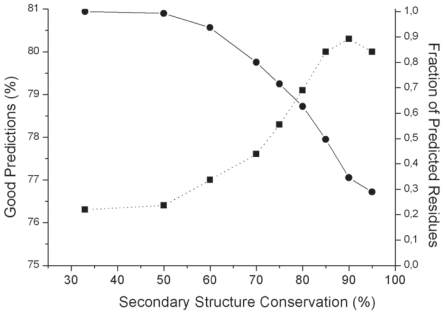
**Prediction of relative solvent accessibility.** Evolution of the level of good predictions (dotted line) and of the fraction of predicted residues (solid line) as a function of the secondary structure conservation.

**Figure S4:**
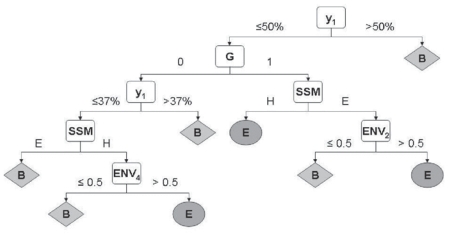
**Prediction of relative solvent accessibility.** Decision tree selected in a two-states model (B = buried, E = exposed) with a threshold of 16%. *G* = *group parameter, ENV* = *environment parameter, SSM* = *secondary structure* − *major state, y_1_* = *topohydrophobic index.*

- **Influence of the work position occupancy on prediction.** To evaluate the influence of the available data in a work position, we used a Q2 index in a two state model with a classical relative solvent accessibility threshold of 16%. Figure S2 shows that, as expected, the level of good predictions increases with the occupancy of a work position and is quite satisfying above 8 to 10 members per work position. For the time being, the bottom level of major secondary structure is kept at 75% for each considered work position, as described in the Material and Methods section.
- **Influence of the major secondary structure threshold on prediction.** We fixed the minimal work position occupancy at 8 and let the major secondarystaterangefrom33%to100%. Figure S3 shows the link between these parameters and confirms that a level of 75% for the major secondary structure threshold constitutes an acceptable compromise for a large-scale study. When work position occupancy and major secondary structure conservation are high, predictions are better but remain applicable to a reduced set of work positions. The couple (1, 33 %) leads to 74% of good predictions for 100% of H or E positions. In contrast, (35, 95%) leads to 87 % of good predictions but only for 3% of H or E positions. (8, 75%) and (10, 80%) give 77% and 79% of good predictions, respectively, for 40% and 27% of H or E positions. All these predictions are performed through a 10-fold cross-validation procedure on the whole bank of 16 000 residues occupying a work position.
- **Two states prediction.** Figure S4 shows the decision tree built for a two-state model with a threshold at 16% of relative solvent accessibility and work positions (10, 80%). It led to 79% of good predictions. Clearly, Env_1_ and Env_3_ are of minor influence with respect to Env2 and Env4 tuned on the natural periodicity of strands and helices, respectively.
- **Three states prediction.** Using the same definition of work positions (10, 80 %) and a three-state model with classical 9% and 36% thresholds of relative solvent accessibility, a similar process leads to a Q3 of 65 good predictions.
- **Comparison with previous approaches.** In order to evaluate the predictive power of our approach, in the case where the secondary structure is assumed to be “ideally” known (i.e. by automatic assignment based on experimental atomic coordinates), we compared it to results obtained on banks composed of between 111 and 421 structures by other sophisticated approaches using neural networks (NN (Rost and Sander 1994), Bayesian statistics (Thompson and Goldstein 1996) and probability profiles/support vector machines (PP) (Gianese et al. 2003)). For example, for a 16% threshold, Q2 are around 75% for NN and PP and around 79% for our approach; for a 9%–36% three states model, Q3 are close to 58% for all methods and around 65% for our approach.

It is worth noting that our approach does not predict all positions (it focuses on available work positions, see Material and Methods for a definition of these work positions) and that it should be less accurate using predicted secondary structures than assigned ones. However, our aim is to provide accurate constraints on a limited set of positions of a fold. Moreover, the performance of our approach would only slightly decrease by using predicted secondary structures, as suggested by its application on four examples reported in Table S3, for which the secondary structures were predicted from multiple alignments using PHD (Rost and Sander, 1994). The decision tree used for prediction is built with the 16 000 positions of the 270 FSSP families, as described above. Our solvent accessibility predictions were then compared to the actual burying level and to the burying state predicted through PHD using the same alignments, in a standard 9%–36% three-state model (exposed, intermediate, buried). The Q3 values calculated on predicted residues are generally better for our approach, called RAPT (Relative Accessibility Prediction Tool) (Table S3). Besides the residues correctly predicted by both methods, the number of residues correctly predicted by RAPT (and not by PHD) is higher than the reverse situation (Table S3). Thus, within work positions, RAPT provides on average better prediction results than PHD. Although addressing a limited number of residues, it takes advantage of simple decision rules, which are easily interpretable.

**Table S3:** **Prediction of relative solvent accessibility with predicted secondary structures.** Three-state prediction (buried/intermediate/exposed; threshold values 9% and 36%). Application to four different families. The SM number in the multiple alignment column refers to the SMART accession number (Letunic et al. 2004). Secondary structures were predicted from these multiple alignments using PHD (Rost and Sander, 1994).

Domains	Multiple alignment	Leader sequence	Number of sequences	Q3 RAPT	Q3 PHD	Number of predicted residues	Consensus RAPT/PHD		No consensus Good Predictions	
							False pred	Good pred	RAPT	PHD
BAH	SM00439 (Callebaut et al. 1999)	Yeast Orc1p (pdb 1m4z)	10	53%	46%	43	14	15	8	5
BRCT	SM00292 (Bork et al. 1997; Callebaut and Mornon 1997)	Human XRCC1 (pdb 1cdz)	15	66%	40%	35	7	11	12	3
ABC NBD	(Callebaut et al. 2004)	Human CFTR (pdb 1xmi)	10	60%	54%	110	14	48	18	12
C2	SM00239	Rat phospholipase C-δ1 (pdb 1dji)	11	63%	58%	52	7	21	12	8

ReferencesBorkPHofmannKBucherP1997A superfamily of conserved domains in DNA damage-responsive cell cycle checkpoint proteinsFASEB. J1168769034168CallebautICourvalinJCMornonJP1999The BAH (bromo-adjacent homology) domain: a link between DNA methylation, replication and transcriptional regulationFEBS Lett446189931010064010.1016/s0014-5793(99)00132-5CallebautIEudesRMornonJP2004Nucleotide-binding domains of human cystic fibrosis transmembrane conductance regulator: detailed sequence analysis and three-dimensional modeling of the heterodimerCell. Mol. Life Sci61230421474550110.1007/s00018-003-3386-zPMC11138792CallebautIMornonJP1997From BRCA1 to RAP1: a widespread BRCT module closely associated with DNA repairFEBS Lett4002530900050710.1016/s0014-5793(96)01312-9GianeseGBossaFPascarellaS2003Improvement in prediction of solvent accessibility by probability profilesProt. Eng159879210.1093/protein/gzg13914983079KabschWSanderC1983Dictionary of protein secondary structure: pattern recognition of hydrogen-bonded and geometrical featuresBiopolymers222577637666733310.1002/bip.360221211LetunicICopleyRRSchmidtS2004SMART 4.0: towards genomic data integrationNucleic Acids Res32D14241468137910.1093/nar/gkh088PMC308822RostBSanderC1994Combining evolutionary information and neural networks to predict protein secondary structureProteins195572806608710.1002/prot.340190108RostBSanderC1994Conservation and prediction of solvent accessibility in protein familiesProteins2021626789217110.1002/prot.340200303ThompsonMJGoldsteinRA1996Predicting solvent accessibility: higher accuracy using bayesian statistics and optimized residue substitution classesProteins253847872731810.1002/(SICI)1097-0134(199605)25:1<38::AID-PROT4>3.0.CO;2-G

## Figures and Tables

**Table 1:** Contacts achieved by strand work positions with topohydrophobic index y_1_ = 1

Absolute number of non-trivial neighbors	Class			
	Alpha	Beta	Alpha/beta	Alpha+beta
Neighbors within helices	0.22	0.04	0.26	0.28
Neighbors within strands	5.04	5.00	5.12	4.87
Neighbors within coils	0.90	0.88	0.88	1.00
Total number of neighbors	6.16	5.92	6.26	6.15
G1 neighbors	4.02	3.25	4.51	3.79
